# Global disease score (GDS) is the name of the game!

**DOI:** 10.1007/s00259-019-04383-8

**Published:** 2019-06-10

**Authors:** Poul F. Høilund-Carlsen, Lars Edenbrandt, Abass Alavi

**Affiliations:** 1 Department of Nuclear Medicine, Odense University Hospital, 5000 Odense C, Denmark; 2 Research Unit of Clinical Physiology and Nuclear Medicine, Department of Clinical Research, University of Southern Denmark, Odense, Denmark; 3 Region Västra Götaland, Sahlgrenska University Hospital, Department of Clinical Physiology, Gothenburg, Sweden; 4 Department of Radiology, Perelman School of Medicine, University of Pennsylvania, Philadelphia, PA USA

For some decennia, CT and MRI have with beautiful images predominated medical in vivo imaging. However, because of their limited sensitivity and structural nature, they have not provided quite the key to improved understanding of disease or better solution to clinical problems that their impressive images promised that they could. Therefore, it is time for molecular imaging in the shape of PET/CT and PET/MRI to utilize the unique advantages of PET and hybrid imaging to accomplish these very important challenges. This requires easily accessible and reliable quantification procedures, which can assess the entire disease burden in the body and provide a simple score expressing the extent and activity of disease within minutes. In this editorial, we argue that the *global disease score (GDS)* is this measure, and must replace the maximal standardized value (SUVmax), which is an easily accessible, but often misleading, oversimplification.

PET was conceived in the late 1950s and came to birth in the 1970s. So did CT and MRI. They shared the same tomographic principle, but because of their higher spatial resolution, CT and MRI for some time put PET on the sideline, until a change began with the emergence of hybrid PET/CT at the turn of the millennium and was reinforced with the advent of PET/MRI approximately 10 years later. The wonderfully sharp images one can achieve with CT and MRI have helped countless patients and thousands of doctors around the world to form an impression of what was wrong and what to rectify. However, the enthusiasm has gradually been damped by the realization that these modalities are hampered by non-negligible limitations, meaning that the tissue changes they depict are typically late-occurring events that seldom appear until cure is no longer an option or lesions no longer represent active disease. Together with their suboptimal sensitivity, these are the main reasons why CT and MRI have not delivered what they were supposed to in the understanding and management of a number of serious disorders including cancer and cerebral, cardiovascular, and musculoskeletal diseases. Thus, it is time for molecular imaging with PET to leave the sideline and step into play.

Besides being molecular and possessing a thousand times higher sensitivity than CT and MRI, PET is an inherently quantitative modality. Achieving optimal utilization of this potential is not straightforward and is something that has still not reached a stage where measurements at different institutions are directly comparable. For PET to become a clinical success in many more diseases, it is necessary to replace easily accessible measures like SUVmax, SUVpeak, and SULmax [1] with conceptually more correct indices of disease. By representing tracer uptake in only a few of the body’s diseased cells, these values are not true indicators of any disease. They disregard the amount, extension, and activity of disease and the fact that many, if not most, diseases are heterogeneous. However, to replace them with other, more comprehensive and more accurate quantities is a challenge, as it requires great computing power and fast, user-friendly, and reproducible computer programs effectively providing proper segmentation and correction for background uptake and partial volume effect, of which the latter plays a major role especially in small lesions [2, 3]. We suggest instead a single number, the GDS, which expresses the amount and extent of disease and its activity. The concept was introduced already in 1993 for FDG-PET imaging of the brain [4], and can be used for the entire body, tumor tissue, or specific organs such as the heart [5–7]. Semi-automated programs enabling the calculation of GDS and other parameters have for some years been used to study dependence on age, gender, and body mass index, for instance, or potential correlation of PET measures with risk factors [8–13]. However, programs are still too time-consuming and somewhat operator-dependent, making them awkward in the daily routine. With the advent of artificial intelligence-based and deep learning procedures, this is about to rapidly change [14–16].

**Fig. 1:**
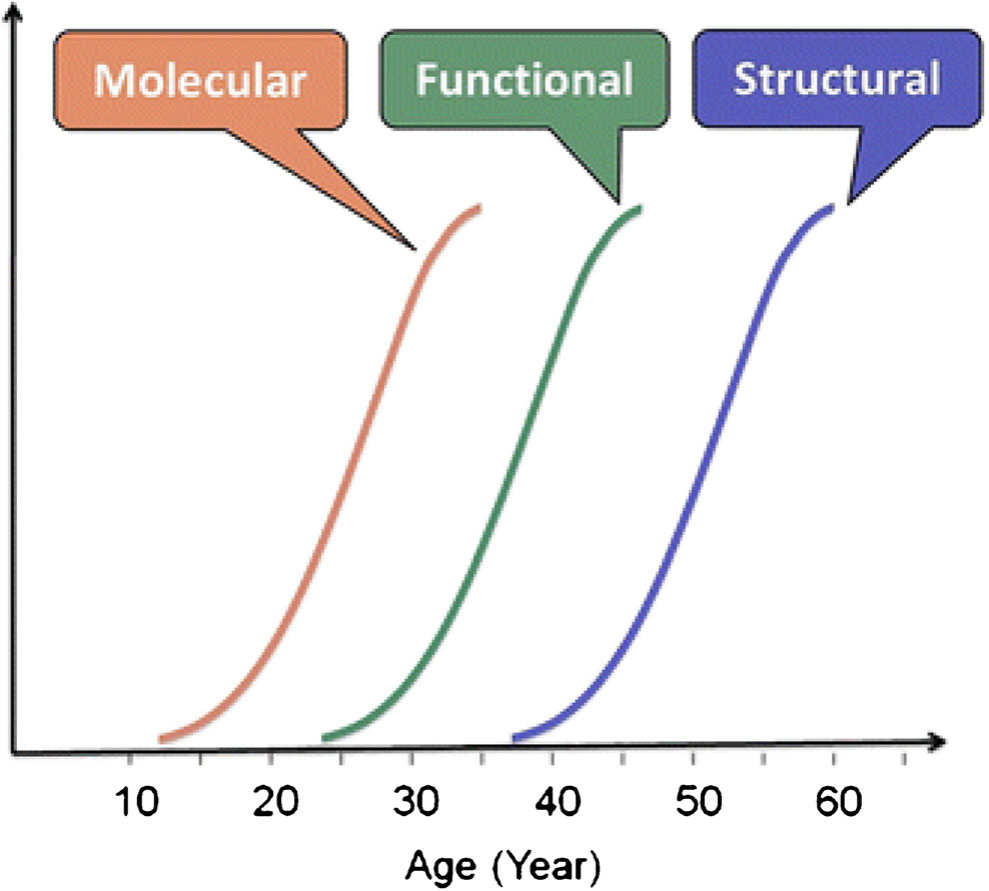
Anticipated temporal relationship between molecular calcification, functional changes, and CT-visible arterial calcification. Reproduced with permission [13]

PET is not a 100% accurate and reliable modality. Its main limitation is a spatial resolution of half a centimeter or more even with modern PET/CT scanners. This cannot be fully counterbalanced by the high sensitivity of PET. It means that a negative PET/CT scan cannot rule out ongoing cancer. However, while a negative FDG PET scan in a patient with a cancer above the size of the spatial resolution of PET is routinely interpreted as false-negative in terms of tumor detectability, it should in fact be interpreted as true-negative in terms of tumor biology, since tumors with low or no FDG uptake are histologically and clinically non-aggressive [17]. Moreover, the high sensitivity of PET and its molecular nature ensures disease detection much earlier than structural imaging, since molecular disease precedes functional disturbances, which in turn appear before structural tissue changes become detectable (Fig. 1). Thus, by its very nature, PET will become positive and demonstrate ongoing disease long before structural imaging will show late consequences of disease that may or may not represent active disease [18].

We are not aware of studies that have examined these challenges in depth. Two recent publications in the *Journal of Nuclear Medicine (JNM)* and *Radiology* based on the same material comprising 34 patients with newly diagnosed high-grade, resectable osteosarcoma illustrate the problem. They describe FDG PET/CT measures obtained at baseline and following 5-week and 10-week neoadjuvant chemotherapy as predictors of histologic response, event-free survival, and overall survival [19, 20]. The JNM report focuses on SUVmax, whereas the *Radiology* article concentrates on SUVpeak, metabolic tumor volume, and total lesion glycolysis, i.e., the product of SUVmean and metabolic tumor volume. In the *JNM* paper, the authors found that SUVmax at 5 weeks and 10 weeks and percentage change in SUVmax from baseline to 10 weeks were highly predictive of histologic response, whereas there was no association with event-free survival, which was not associated with either change or percentage change in SUVmax [19]. In the *Radiology* article, the authors found that SUVpeak, metabolic tumor volume, and total lesion glycolysis measured at baseline were predictors of event-free survival and overall survival, and that at 5 weeks and 10 weeks, all parameters including percent change were associated with histologic response and predictive of event-free survival [20]. By its definition, SUVmax cannot represent an entire tumor or the total burden of cancer in the body. Consequently, it is no surprise that parameters derived instead from the whole primary tumor and not a tiny fraction of it were predictive of event-free and overall survival and were more reliable endpoints of response to chemotherapy than histology, which was only borderline significantly predictive [20].

Because SUVmax represents the uptake in just a single voxel and is influenced by noise, movement artefacts, and other sources of error, it is not a relevant measure of a disease and its development. Like many other cancers, osteosarcoma very heterogeneous [21]. As expressed by Lindsey et al. it is “extremely heterogeneous in both its origins and manifestations” and “continues rapidly modifying its genotype, thus making potential targeted molecular therapeutics increasingly impractical” [22]. For the same reason, more specific PET tracers than FDG for cancer imaging are a rare sight. In the *Radiology* report, metabolic tumor volume was as predictive as total lesion glycolysis, perhaps partly due to the chosen tumor type (high-risk and surgically removable), which could mean less heterogeneity than if all histological degrees were included. Thus, it is fair to assume that total tumor glycolysis, or rather the GDS, which is the weighted average uptake in all lesions, is a truer indicator of the amount and aggressiveness of the tumor burden than just the tumor volume, the SUVmax, or the diameter measures that are still retained in the Response Evaluation Criteria in Solid Tumors (RECIST) criteria [23]. Heterogeneity exists and changes spontaneously within the tumor volume, in particular when chemotherapy and other interventions kill or subdue part of a primary tumor. The Positron Emission Tomography (PET) Response Criteria in Solid Tumors (PERCIST) criteria was a step in the right direction by shifting from a geometric to an uptake measure [24]. Unfortunately, it suggests measurement of SULpeak in the hottest lesion and potentially also in five selected lesions, which are the same from time to time. Since these will typically change and thus retain their characteristics for only a limited period due to ongoing genetic and phenotypic changes, the risk of misinterpretation increases with the rate of these chances.

**Fig. 2:**
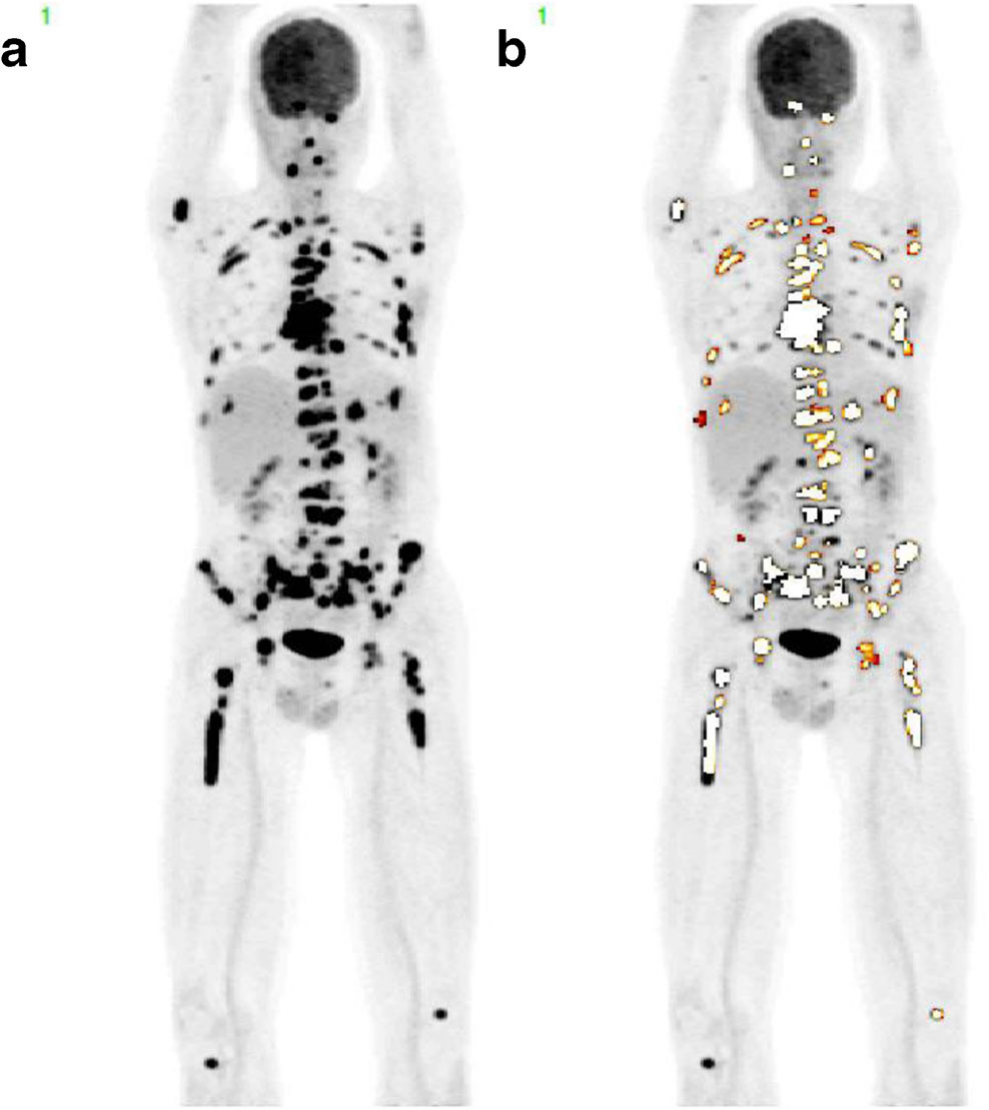
**a** FDG-PET scan of a patient with multiple myeloma and osseous and extra-osseous lesions. **b** Lesions were analyzed using dedicated semi-quantitative software. Segmentation was done using a voxel-based iterative adaptive algorithm starting at 40% threshold of SUVmax. Note how difficult it is to determine which lesions should be included in the analysis and how difficult it might be for different observers to achieve the same result. Artificial intelligence-based analysis overcomes these challenges. Courtesy Dr. Brian Østergaard, Department of Hematology, Odense University Hospital, Odense, Denmark

**Fig. 3:**
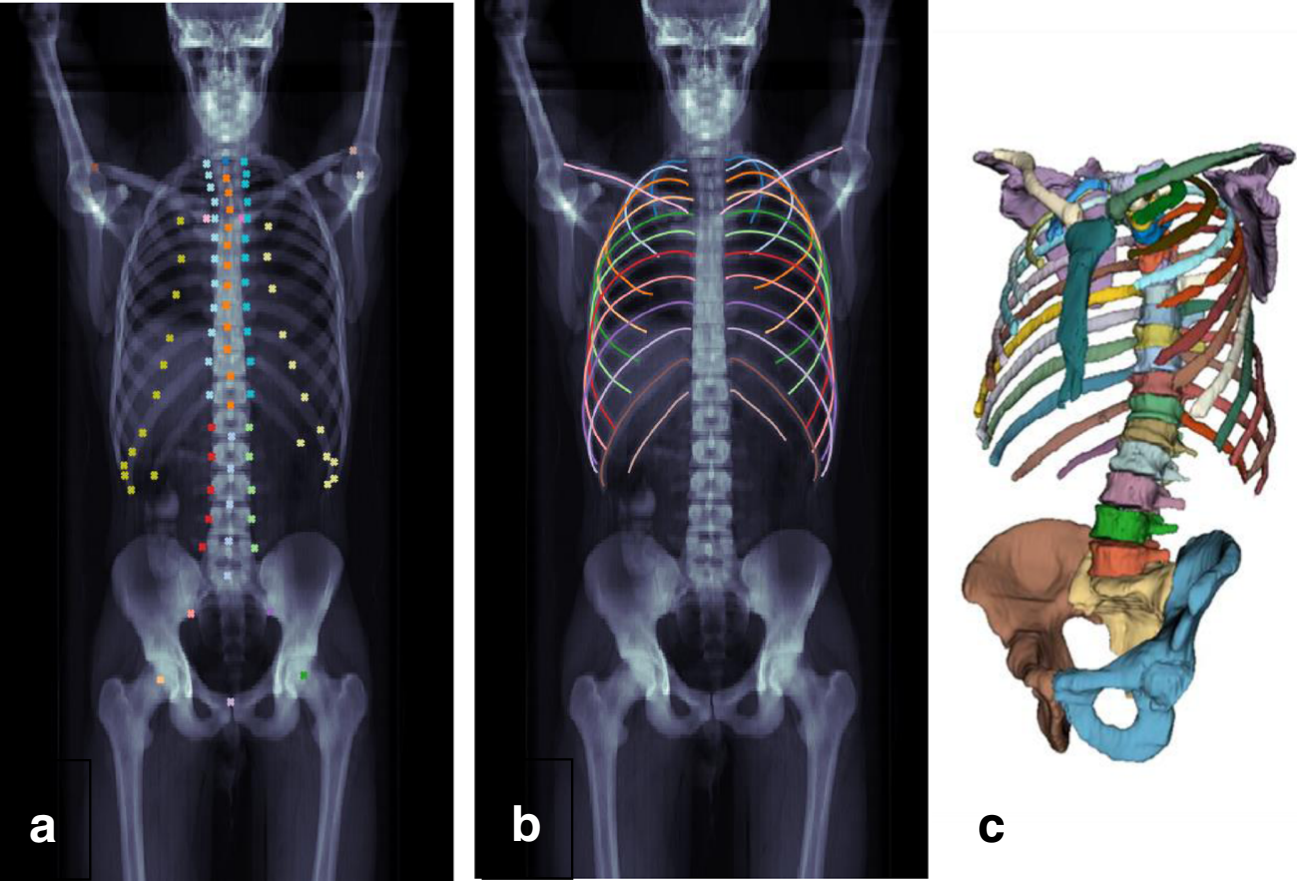
**a** Maximum-intensity projection of a CT scan with annotated landmarks. **b** Detected center lines for ribs and clavicles. **c** Surface reconstruction of the resulting segmentation enabling calculation of the skeletal volume in relation to which the sum of the metabolically active volume of bone lesions is expressed. Reproduced with permission [15]

Only the entire burden of disease, its extent, and activity can truthfully characterize and monitor the disease and its response to treatment. That is exactly what is expressed by the GDS. To what extent this score should be supplemented with measurements of the rate of change in disease activity is a question to be highlighted in future research. The rationale behind double-time-point PET imaging using for instance 1- and 3-h acquisition is readily understandable, because this will reveal the degree of tumor aggressiveness [25]. In practice, however, this approach is cumbersome, and its ability to characterize the disease stage of individual patients may be somewhat limited [26, 27]. Another option is trend analysis based on repeat PET acquisitions, except that this requires three or more acquisitions, i.e., one at baseline and two or more during follow-up [28]. In addition, good reproducibility is absolutely necessary, since all involved measurement errors come into play twice when the measurement is repeated [29]. Manual methods for quantifying total disease volume and activity are too time-consuming and hampered by low reproducibility (Fig. 2). The use of modern artificial intelligence-based methods for single and repeat GDS measurements makes sense, because these can be obtained in a completely automated and operator-independent way within minutes or seconds (Fig. 3), which means that they can be applied and their performance validated much more rigorously. Importantly, the AI-based approach allows for the first time for comparison of GDS results obtained at different institutions with different vendor-specific scanners, reconstruction algorithms, and processing procedures, and that is exactly what is required in the clinical context and for research purposes. Objectivity and reproducibility are critical according to the FDA biomarker qualification review team, which states that the clinical association of a quantitative biomarker is significant only if the marker is measured consistently under pre-defined conditions [30], which again is a prerequisite for performing analytical and clinical validation studies such as those conducted recently for the Bone Scan Index [31].

In conclusion, the time has come to abandon illogical and misleading PET imaging measures representing but a fraction of vastly heterogeneous diseases in the body, in favor of ultra-fast, operator-independent, highly reproducible AI-based measurements of the GDS, which is a much truer expression of the body’s disease load that is constantly undergoing spontaneous or therapy-induced changes.
